# Monitoring Ion Activities In and Around Cells Using Ion-Selective Liquid-Membrane Microelectrodes

**DOI:** 10.3390/s130100984

**Published:** 2013-01-15

**Authors:** Seong-Ki Lee, Walter F. Boron, Mark D. Parker

**Affiliations:** Department of Physiology and Biophysics, School of Medicine, Case Western Reserve University, 10900 Euclid Avenue, Cleveland, OH 44106, USA; E-Mails: sxl271@case.edu (S.-K.L.); wfb2@case.edu (W.F.B.)

**Keywords:** ISM, LIX, Nernst equation

## Abstract

Determining the effective concentration (*i.e.*, activity) of ions in and around living cells is important to our understanding of the contribution of those ions to cellular function. Moreover, monitoring changes in ion activities in and around cells is informative about the actions of the transporters and/or channels operating in the cell membrane. The activity of an ion can be measured using a glass microelectrode that includes in its tip a liquid-membrane doped with an ion-selective ionophore. Because these electrodes can be fabricated with tip diameters that are less than 1 μm, they can be used to impale single cells in order to monitor the activities of intracellular ions. This review summarizes the history, theory, and practice of ion-selective microelectrode use and brings together a number of classic and recent examples of their usefulness in the realm of physiological study.

## 1. Introduction

The activity of an ion is the effective concentration of that ion in a mixture of chemicals. This value is generally slightly smaller than the molar concentration of the parent chemical species because some salts do not fully ionize in solution. Moreover, the extent of ionization can be influenced by the presence of other chemical species in the solution as well as by factors such as temperature and ionic strength. Importantly it is the activity (*a*), rather than the concentration, of an ion that determines the thermodynamic contribution of the ion to a system such as membrane potential (a parameter that is predominantly determined by the intracellular and extracellular activities of Na^+^, K^+^, and Cl^−^) and the rate of chemical reactions in which physiologists are interested. Every cell has the ability to control the distribution of ions across its membranes by virtue of the channels and transporters that are present in each membrane.

Three classical technologies that are applied to determining ion activity/concentration and monitoring the movement of ions across cell membranes are radiolabeled tracers (e.g., reference [1]), ion-sensitive fluorescent indicator dyes [2], and ion-selective microelectrodes (ISMs). ISMs based on ion-selective liquid-membranes are the focus of the present review. These ISMs are glass microelectrodes that are used to continuously monitor the activity of a specific ion at a specific locus by virtue of their tips being filled with an ion-selective liquid membrane. ISM use facilitates ion measurement because

1. Numerous ISMs can be applied to a single cell at the same time, allowing numerous ion activities to be monitored simultaneously.
2. ISMs can be applied to monitor ion activity at specific loci such as the cell surface or the cytoplasm.
3. The reference electrode that is paired with the ISM for measurement of intracellular ion activity (see Section 5) provides a simultaneous measurement of membrane potential providing a more complete characterization of the transport processes that contribute to the changes in ion activities.
4. In combination with vibrating probe technology [3], ISMs can be used to measure net ion fluxes.

However, ISMs also have disadvantages:

1. Ionophore-doped liquid membranes are imperfectly ion-selective.
2. The use of ISMs to monitor intracellular ion activities is best applied to large cells that can be easily impaled with a microelectrode (e.g., *Xenopus* oocytes, which have a diameter that is greater than 1 mm; approximately 50–100 times larger than a typical mammalian cell).

The earliest ISMs were fabricated from ion-selective glass [4] but their usefulness for intracellular ion-measurements, which requires impalement of a cell, is limited by their relatively large tip diameter, slow response time, and expertise required to fabricate them [5]. However their worth is evidenced by studies of, for example, pH_i_ regulation in snail neurons [6], giant-barnacle muscle-fibers [7], and squid axons [8] that were conducted using ISMs based on H^+^-selective glass.

The replacement of an ion-selective glass tip with an ionophore-doped liquid membrane (also known as an ionophore cocktail) conferred ion selectivity to glass microelectrodes with a smaller (less than 1 μm) diameter tip and a *t*_90_—the time taken for 90% of the full electrode response to occur—on the order of seconds (see reference [9] for a review of more recently recommended measures of ion-selective electrode response times). Here, we discuss the theory, fabrication, and application of ionophore-cocktail based ISMs. The theory, construction, and application of ISMs have also been reviewed by others in references [4,10–13].

## 2. Theory of ISMs

In order for an ISM to be useful, the microelectrode must respond predictably and rapidly to changes in ion activity such that the voltage reported by the microelectrode can be used to recreate information about ion concentration. Underlying the theoretical considerations that describe the relationship between the electrical signal reported by an ISM and the ionic composition of the solution to which the ISM is exposed are the concepts of ion activity and electrochemical potential. In the following subsections, we first consider the theoretical behavior of gases in a closed system and extend that theory to uncharged solutes and ions. We then consider the distribution of a single ion species across a semi-permeable membrane, and finally the electrochemical potential difference across an ion-selective liquid membrane such as an ionophore cocktail.

### 2.1. Gibbs Energy

Gibbs energy (*G*) is the potential energy that can be absorbed or released during a chemical reaction in a closed system. *G* is a function of internal energy (*U*), pressure (*P*), volume (*V*), temperature (*T*), and entropy (*S*) as shown in Equation (1):

(1) G=U+PV−TS.

Equation (1) can be differentiated to:

(2) dG=dU+PdV+VdP−TdS−SdT.

Equation (2) can be simplified because *dU* = *dQ* − *PdV* (the first law of thermodynamics, in which Q is heat) and *dQ* = *TdS* (the second law of thermodynamics). Thus at fixed temperature (*dT* = 0), Equation (2) can be restated as:

(3) dG=VdP.

Substituting the definition of *V* into Equation (3) according to the ideal gas law (*V* = *nRT/P*), where *R* is the ideal gas constant, describes the relationship between *G* and *P*:

(4) $$dG=\frac{\text{nRT}}{P}dP.$$

*dG* is defined as an infinitesimally small change in G in Equations (2), (3), and (4). But if we assume that our model system changes from an initial state “1” to a final state “2”, causing a measureable change in G (Δ*G* = *G*_2_ − *G*_1_), we can integrate Equation (4) between states 1 2, as shown below in Equation (5). (Note that ∫(*dP/P*) = ln *P*):

(5) $$\Delta G=\int_{1}^{2}dG=G_{2}-G_{1}=\int_{1}^{2}\frac{\text{nRT}}{P}=dP=\text{nRT}\int_{1}^{2}\frac{dP}{P}=\text{nRT}(\ln P_{2}-\ln P_{1})=\text{nRT}\ln\frac{P_{2}}{P_{1}}.$$

That is to say:

(6) $$G_{2}=G_{1}+\text{nRT}\ln\frac{P_{2}}{P_{1}}.$$

Defining the initial state 1 as a standard state where *G*^⊖^ is the standard free energy and *P*^⊖^ is the standard state pressure (1 atm), provides us with Equation (7):

(7) $$G=G^{\Theta }+\text{nRT}\ln\frac{P}{P^{\Theta }}.$$

In our closed system (at constant *T*, *V*) Equation (7) can be restated in terms of chemical potential, (*μ*) which can be described as Gibbs energy per mole *(G/n)*:

(8) $$\mu =\mu^{\Theta }+RT\ln\frac{P}{P^{\Theta }}.$$

### 2.2. Chemical Potential and Activity

In the case of a solution, it is more useful to consider the concentration of a molecule ([X]) rather than its pressure (*_P_*). Henry's law relates pressure and concentration by a constant *k_H_* that cancels out when we substitute the equation *P* = *k_H_* [*X*] into Equation (8):

(9) $$\mu =\mu^{\Theta }+RT\ln\frac{\left[X\right]}{{\left[X\right]}^{\Theta }}.$$

Because the standard state concentration of particle [*X*] ^⊖^ = 1 M, this term does not need to be included in the equation. As we noted earlier, because of the influence of physical factors upon solubilization, the activity of a chemical species (*a*) is usually less than its molar concentration [14]. Thus we need to modify the relationship with an activity co-efficient (*γ*) which relates the activity and concentration of a species (*γ* [*X*] = *a*) such that:

(10) $$\mu =\mu^{\Theta }+RT\ln\gamma [X]=\mu^{\Theta }+RT\ln a.$$

### 2.3. Electrochemical Potential

In order to extend our consideration to charged particles (*i.e.*, ions) we must further modify Equation (10) to describe the contribution of charge to the electrochemical potential (*μ̃*) of the system, where *z* is the valence of the ion, *F* is Faraday's constant, and Ψ is electrostatic potential of the system:

(11) $$\tilde{\mu }=\mu^{\Theta }+RT\ln a+zF\Psi .$$

Note that, if a particle is uncharged, *z* = 0 and *μ̃* = *μ*, per Equation (10).

### 2.4. Electrochemical Potential Difference across a Semi-Permeable Membrane

**Figure 1.**
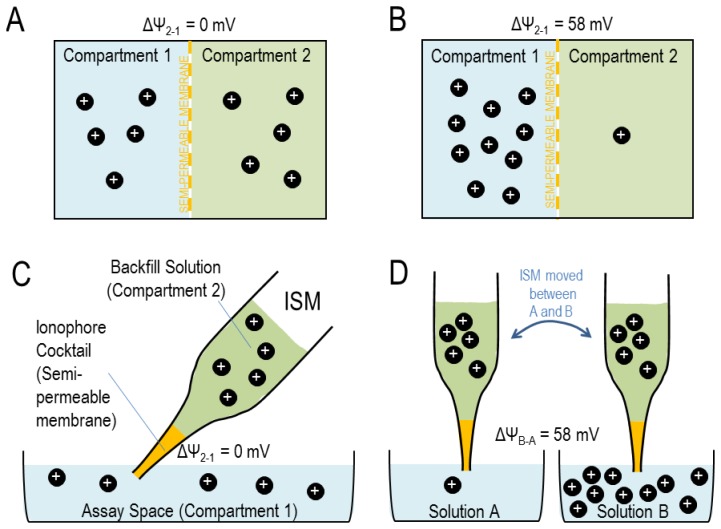
(**a**) Two compartments that contain equal numbers of ions, separated by a semi-permeable membrane. (**b**) Two compartments that differ ten-fold in ion content, separated by a semi-permeable membrane. (**c**) Model of an ISM based on Figure 1(A). (**d**) Model of ISM calibration.

Now, let us imagine two compartments 1 and 2 that are separated by a selectively-permeable barrier such as a cell membrane or an ionophore cocktail that permits selective permeation of a certain ion (shown in Figure 1, panels A and B).

When the two compartments are at equilibrium (*i.e.*, Δ*G* = 0), net ion movement across the membrane is zero and the electrochemical potentials are identical between the two compartments, such that *μ̃*_1_ = *μ̃*_2_. In this case, the electrical potential difference at equilibrium can be described as:

(12) $$\mu^{\Theta }+RT\ln a_{1}+zF\Psi_{1}=\mu^{\Theta }+RT\ln a_{2}+zF\Psi_{2}$$

or:

(13) $$zF(\Psi_{2}-\Psi_{1})=RT\ln\frac{a_{1}}{a_{2}}$$

(14) $$\Delta \Psi_{2-1}=\frac{RT}{zF}\ln\frac{a_{1}}{a_{2}}.$$

Because *a* = *γ* [X^z+^] (or [X^z-^] in the case of an anion) and assuming that the physiochemical properties of the two compartments are similar such that *γ*_1_ is approximately the same as *γ*_2_, we derive the Nernst equation:

(15) $$\Delta \Psi_{2-1}=\frac{RT}{zF}\ln\frac{{\left[{\text{X}}^{z+}\right]}_{1}}{{\left[{\text{X}}^{z+}\right]}_{2}}.$$

At room temperature, *RT*/(*F* × log*e*) = 0.058 thus:

(16) $$\Delta \Psi_{2-1}=\frac{0.058}{z}\log\frac{{\left[{\text{X}}^{z+}\right]}_{1}}{{\left[{\text{X}}^{z+}\right]}_{2}}.$$

That is to say, when both compartments contain an equal concentration of [X^z+^], ΔΨ_2−1_ = 0 mV (Figure 1(A)) but a ten-fold difference in [X^z+^] across an X^z+^-permeable membrane will produce an electrical potential difference across that membrane of 0.058 V = 58 mV in the case of a monovalent ion (Figure 1(B)), or 29 mV in the case of a divalent ion. This predictable electrical response to changes in ion activity is the basis for the usefulness of ISMs.

### 2.5. Electrochemical Potential Difference across an Ion-Selective Liquid Membrane

In the case of an ISM, we can consider the ionophore cocktail in the tip of the electrode as the semi-permeable membrane. A ‘backfill’ solution within the ISM (Figure 1(C); equivalent to compartment 2 in Figures 1(A) and 1(B)) provides the electrical connection between a silver wire (Ag/AgCl half-cell) in the microelectrode holder (see Section 5.1) and the ionophore cocktail. The assay space into which the electrode tip is placed, of which we would like to know the ion concentration (Figure 1(C)), is equivalent to compartment 1 in Figures 1(A) and 1(B). Thus a ten-fold concentration difference between [X^z+^] in the assay space and [X^z+^] in the backfill solution is registered as a 58/*z* mV potential difference across the cocktail.

Crucially, given that the backfill solution in compartment 2 has a fixed composition ([X^z+^] *_backfull_*) and assuming that the activity co-efficient for [X^z+^] is constant in the assay space, we can determine that the potential difference between two solutions A and B containing [X^z+^]*_solutionA_* and [X^z+^]*_solutionB_* is:

(17) $$\Delta \Psi_{\text{solutionB}-\text{solutionA}}=\frac{0.058}{z}\left(\log\frac{{\left[{\text{X}}^{z+}\right]}_{\text{solutionB}}}{{\left[{\text{X}}^{z+}\right]}_{\text{backfull}}}-\log\frac{{\left[{\text{X}}^{z+}\right]}_{\text{solutionA}}}{{\left[{\text{X}}^{z+}\right]}_{\text{backfull}}}\right)=\frac{0.058}{z}\log\frac{{\left[{\text{X}}^{z+}\right]}_{\text{solutionB}}}{{\left[{\text{X}}^{z+}\right]}_{\text{solutionA}}}.$$

**Figure 2.**
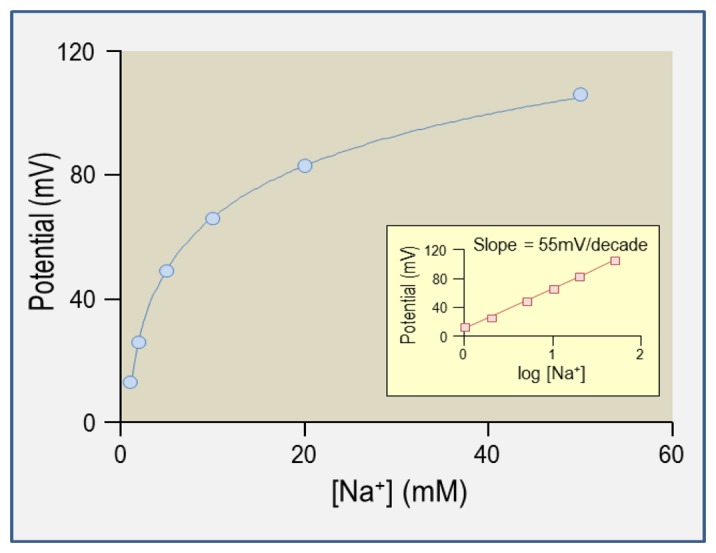
Example calibration plot for an intracellular Na^+^-selective microelectrode gathered using NaCl solutions.

That is to say, a ten-fold concentration difference between solution A and solution B will register as a 58 mV potential difference between the two solutions for a monovalent ion [Figure 1(D)]: a Nernstian response. The exhibition of a Nernstian response by an ISM proves that it is selectively permeable to the ion of interest among all ions present in the solutions. This is the basis of calibration, an example of which is shown in Figure 2. Practical aspects of electrode calibration are considered in Section 5.3. Traditionally, K^+^- and H^+^-selective ISMs are more ideally ion-selective than Na^+^- or Cl^−^-selective ISMs. A consideration of the selectivity of each cocktail is presented in Sections 3.1 to 3.4 (guidelines for determining the selectivity coefficients for ISMs are provided in reference [15]). Note that the activity of Na^+^ decreases as [Na^+^] increases causing the calibration slope of Na^+^-selective ISM in Figure 2 to be slightly less than the Nernstian ideal of 58 mV/decade. Note that the estimated activity coefficients of the ions considered in this review (H^+^, Na^+^, K^+^, and Cl^−^), are 0.98 at 1 mM, 0.93 at 10 mM, and 0.82–0.86 at 100 mM [14].

## 3. The Composition of H^+^, Na^+^, K^+^, and Cl^−^ Ionophore Cocktails and Backfill Solution

In this section we consider the chemical components of frequently used, commercially-available ionophore-cocktails and the associated backfill solutions that are used in the manufacture of ion-selective microelectrodes. Ionophore cocktails have three basic components: an ionophore, a water-immiscible solvent for the ionophore that does not substantially affect the ion-selectivity of the ionophore, and additives that are used to modify the electrical resistance, hydrophobicity, viscosity, and ion-selectivity of the cocktail. Ionophore cocktails are commercially available for a variety of other molecules besides H^+^, Na^+^, K^+^, and Cl^−^ (see The Sensor Application Portal hosted at www.sigmaaldrich.com/analytical-chromatography/analytical-reagents/sensoric-applications.htm). The imperfect selectivity of some ionophores means that under certain conditions ion-selective cocktails can be repurposed to monitor changes in the concentrations of “interfering” ions. For example, “Na^+^-selective”-cocktail based ISMs have been used to monitor [Li^+^] (see Section 6).

Backfill solutions, in order to fulfill the theoretical requirements mentioned in the previous section, must have a similar activity coefficient for X^z+^ to the assay space, maintain a constant activity of X*^z^*^+^ throughout the measurement period, and must not contain interfering ions that compromise the selectivity of the ionophore cocktail.

### 3.1. H^+^-Selective Ionophore Cocktails

There are three commercially-available cocktails recommended for use in H^+^-selective microelectrodes: Ionophore I/Cocktail A (cat. no. 95291, Sigma Aldrich, St. Louis, MO, USA), Ionophore I/Cocktail B (cat. no. 95293, Sigma), and Ionophore II/Cocktail A (cat. no. 95297, Sigma). All three cocktails respond linearly to changes in pH over the range 5.5–9.0, which covers most physiological applications. Outside of this range, care must be taken with cocktail choice; cocktails incorporating ionophore I can also be used at more alkaline pH values (up to pH 11, but not below pH 4.5) while cocktails incorporating ionophore II can be used at more acidic pH values (as low as pH 2.0 not above pH 9.0).

Ionophore I is tridodecylamine (TDDA), a lipophilic amine that acts as a proton carrier. In our laboratory we routinely use ISMs containing Ionophore I/Cocktail B (see Table 1) to monitor intracellular and extracellular pH. Cocktails A and B differ only in the identity of the cocktail additive inasmuch as Cocktail A contains sodium tetraphenylborate (NaTPB) rather than potassium tetrakis (4-chlorophenyl)borate (KTCPB). Both cocktails behave essentially the same, both being extremely selective for H^+^ over other ions such as Na^+^ in the physiological range (greater than 10^12^-fold preference) and both exhibiting similar response times. The authors who first reported the use of ISMs based on TDDA recommended overnight incubation of cocktail A with 100% CO_2_ prior to use [16]. This procedure was presumably a precaution to minimize drift of potential due to CO_2_ interference when the ISM is used in biological systems. However, CO_2_ does not interfere appreciably with Cocktail B due to the substitution of the NaTPB additive with KTCPB [12]. Notably, a modified ISM that is fabricated with a CO_2_-permeable column of Ionophore I and a backfill that contains carbonic anhydrase can be used in concert with a H^+^-selective ISM to monitor pCO_2_ [17].

Ionophore II/Cocktail A is composed of the same solvent and additive as Ionophore I/Cocktail B, albeit in a slightly different ratio, but is based on a different H^+^ ionophore, namely 4-nonadecylpyridine (CAS No. 70268-36-9), which has a slightly poorer cation selectivity.

### 3.2. Na^+^-Selective Ionophore Cocktails

There are two commercially-available cocktails recommended for use in Na^+^-selective microelectrodes: Ionophore I/Cocktail A (cat. no. 99314; Sigma, see Table 2) and Ionophore II/Cocktail A (cat. no. 99357; Sigma).

Ionophore I is N,N′,N″-triheptyl-N,N′,N″-trimethyl-4,4′,4″-propylidynetris(3-oxabutyramide)—also known as ETH227—is reasonably selective for Na^+^ over K^+^ (more than 50-fold preference, see reference [19]) but not well selective for Na^+^ over Ca^2+^ (Ionophore I actually exhibits a 1.6-fold preference for Ca^2+^ over Na^+^). Thus Ionophore I/Cocktail A is the component of choice for monitoring of Na^+^ in the cytoplasm [19] in which compartment [Na^+^] is ∼4 mM, [K^+^]∼100 mM, and [Ca^2+^] is <1 μM.

Ionophore II is N,N′-dibenzyl-N,N′-diphenyl-1,2-phenylenedioxydiacetamide, which is also known as ETH157 exhibits a worse selectivity for Na^+^ over K^+^ (5-fold) than Ionophore I, but a better selectivity for Na^+^ over Ca^2+^ (20-fold). Ionophore II/cocktail A is better suited for inclusion in ISMs designed for extracellular measurements in which compartment [Na^+^] may be ∼100 mM, [K^+^]∼3 mM, and [Ca^2+^] ∼2 mM [20].

### 3.3. K^+^-Selective Ionophore Cocktails

There are two commercially-available cocktails recommended for use in K^+^-selective microelectrodes: Ionophore I/Cocktail A (cat. no. 60031; Sigma, see Table 3) and Ionophore I/Cocktail B (cat. no. 60398; Sigma). Ionophore I is valinomycin, which carries K^+^ across membranes [21]. Both cocktails are similar in composition but cocktail A contains the solvent dibutyl sebacate, which confers upon the cocktail an improved selectivity for K^+^ over Na^+^.

### 3.4. A Cl^−^-Selective Ionophore Cocktail

At the time of writing, there is only one commercially available cocktail recommended for use in Cl^−^-selective microelectrodes: Ionophore I/Cocktail A (cat. no. 99408; Sigma, see Table 4). This cocktail is about 30-fold more selective for Cl^−^ over HCO_3_^−^ [25], but is considered poorly selective with regard to its target ion compared to ion-selective cocktails for H^+^, Na^+^, and K^+^. The presence of HCO_3_^−^ at close-to-physiological concentration does not substantially interfere with the Nernstian response of the electrode to Cl^−^, but if the electrode is to be used in the presence of HCO_3_^−^, it should be calibrated in the presence of HCO_3_^−^ in order to correct for interference [26]. Great care should be taken to account for interference by HCO_3_^−^ when designing and interpreting the results of experiments that employ this cocktail, especially (1) if used in an assay space with an alkaline pH (above pH 7.6, see reference [25]), (2) if used in compartments such as the cytoplasm that could contain interfering anions that are beyond the investigators control, or (3) if [HCO_3_^−^] is expected to change substantially over the assay period.

## 4. Fabricating ISMs

### 4.1. Pulling Glass Microelectrodes from Capillary Glass

**Figure 3.**
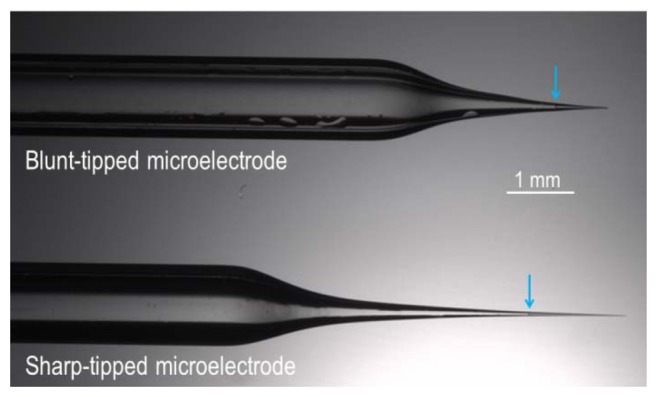
Micrograph of blunt-tipped and sharp-tipped microelectrodes taken with a M205A Stereomicroscope (Leica Microsystems, Buffalo Grove, IL, USA). Blue arrows show ionophore-cocktail meniscus.

Glass microelectrode can be fabricated with either a sharp end (tip diameter∼1 μm) for intracellular recordings or a ‘blunt’ end (tip diameter∼20 μm) for cell-surface/extracellular recordings. We pull our microelectrodes from borosilicate capillary glass using a micropipette puller (for example, we use a Model P-97 Flaming/Brown from Sutter Instrument, Novato, CA, USA). An example of each type of electrode is shown in Figure 3.

Sharp-ended intracellular microelectrodes can be pulled from thin-walled borosilicate glass capillary that contains a filament (we use OD = 2 mm, ID = 1.56 mm; Part No. 30-0077; Harvard Apparatus, Holliston, MA, USA). The usefulness of a batch of pulled microelectrodes can be tested by filling one sample electrode with saturated KCl; the resistance of this KCl-filled microelectrode should be ∼0.5 MΩ. Smaller tip diameters can be achieved by controlled tip-breakage of occluded microelectrodes [29].

Blunt-ended extracellular microelectrodes can be pulled from thick-walled borosilicate glass capillary that contains a filament (we use OD = 2 mm, ID = 1.16 mm; Part No. G200F-4; Warner Instruments, Hamden, CT, USA). The tips of these pulled capillaries are fire polished using a microforge to eliminate sharp edges. The use of thick-walled, fire polished electrodes is important if the ISM is intended to be pushed up against a cell surface or into a tissue slice without cell impalement.

### 4.2. Baking and Silanizing Empty Microelectrodes

Prior to filling with ionophore cocktail, the empty glass microelectrodes must be baked, silanized, and cured [30]. This process makes the inside surface of the microelectrode hydrophobic, which encourages an unbroken interface between the hydrophobic ionophore cocktail and the glass, eliminating shunt pathways between the assay space and backfill solution that circumvent the cocktail and thereby reduce the selectivity of the ISM.

Empty microelectrodes are mounted in a metal rack with their tips pointing upwards and baked overnight in an oven at 200 °C to remove moisture. The following day, still at 200 °C, the microelectrode-containing rack is transferred to a glass petri-dish and covered with an inverted glass-jar to create a loosely sealed glass chamber around the baked microelectrodes. 90 μL of bis(dimethylamino)dimethylsilane (an organic silicon compound; cat. no. 14755; Sigma) is introduced under the jar such that the microelectrodes are exposed to “silane” vapor for 30-50 min within the chamber. Finally the jar is removed and the silane-coated microelectrodes are left to cure at 200 °C overnight. Cooling the cured electrodes to room temperature can be performed in a nitrogen-filled desiccation-chamber containing phosphorous pentoxide, to prevent condensation forming inside the microelectrode tip that could adversely affect ISM performance.

### 4.3. Filling and Backfilling the Microelectrodes

A short column (∼1 mm) of ionophore-cocktail can be introduced into the silanized microelectrode tip either by backfilling (for this purpose we use a 34 Gauge MicroFil™ flexible plastic syringe needle, cat. no. MF34G-5, World Precision Instruments Inc., Sarasota, FL, USA). The filament in each microelectrode draws the ionophore-cocktail into the extremity of the tip (the meniscus of the column of cocktail in each is indicated with cyan arrows in Figure 3). The backfill is introduced beneath the surface of the ionophore cocktail—to avoid introducing an air gap between the backfill and the cocktail—using a wider gauge needle (we use a 28 Gauge MicroFil™ needle, cat. no. MF28G-5, World Precision Instruments Inc.). An alternative approach is to frontfill the electrode, using suction to draw “backfill” solution into the electrode barrel through the tip and then to frontfill the electrode with a short column of ionophore cocktail (e.g., see reference [31]).

## 5. Use and Calibration of ISMs

### 5.1. Mounting ISMs

The microelectrode can be mounted in a straight holder with a female connector jack, a silver wire (Ag/AgCl) half-cell that maintains a constant electrochemical potential within the ISM backfill, and a vent or pressure port (e.g., cat. no. MEH2SFW, World Precision Instruments Inc.).

**Figure 4.**
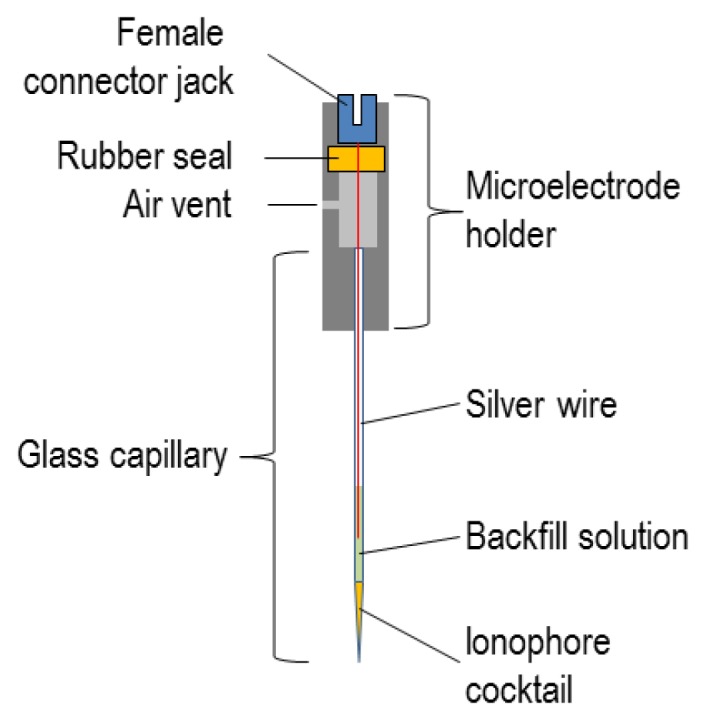
Schematic of microelectrode holder/ISM assembly.

In the final assembly, the ionophore-cocktail column should be a few hundred μm deep and the backfill should be reasonably shallow (perhaps 1 cm), but still deep enough to make contact, when mounted, with the Ag/AgCl half-cell, as shown in Figure 4. The connector jack enables the ISM assembly to be mounted into the electrometer headstage probe (see Section 5.2).

### 5.2. Electrical Set-Up

As discussed in Section 2.5, the ion-selective potential measured by an ISM is described by the equation:

(18) $$\Delta \Psi_{\text{solutionB}-\text{solutionA}}=\frac{0.058}{z}\ln\frac{{\left[{\text{X}}^{z+}\right]}_{\text{solutionB}}}{{\left[{\text{X}}^{z+}\right]}_{\text{solutionA}}}$$

**Figure 5.**
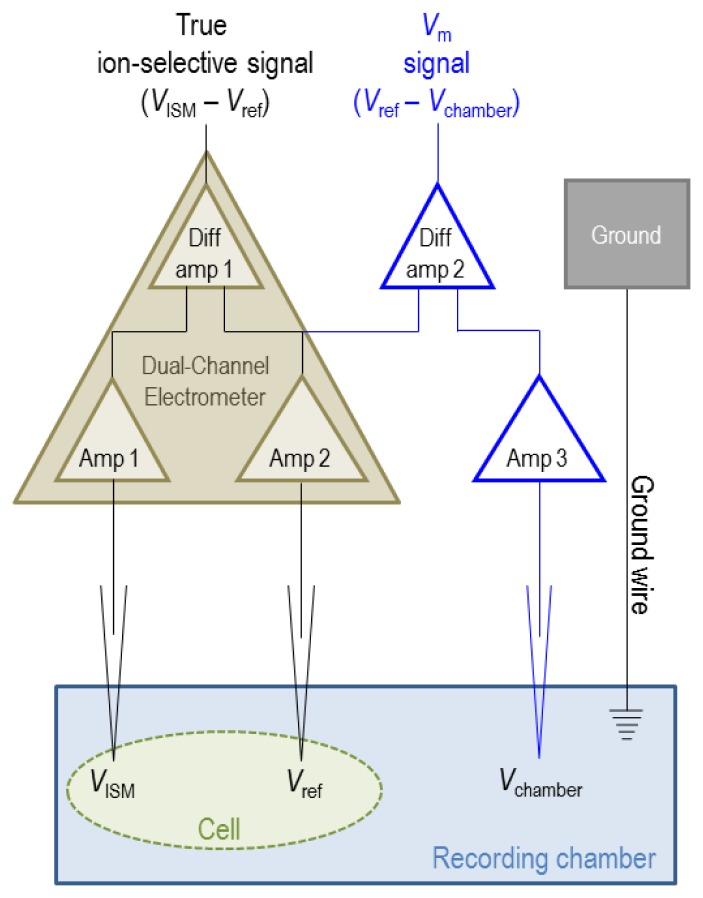
Schematic of electrical configuration required to monitor the ion-selective response of an ISM (brown circuit). Amp = Amplifier, Diff amp = Differential Amplifier. The blue circuit represents optional components required to simultaneously monitor membrane potential (*V*_m_) during intracellular recordings. Faraday shielding is also necessary, but not shown.

Implicit in Equation (18) is the assumption that solutions A and B have equal electrical potentials. In practice, ΔΨ*_solutionB_*_−_*_solutionA_* measured by the ISM can also include a contribution from the difference in electrical potential between solution A and solution B (for example if solution A is the cytoplasm and solution B is extracellular). For this reason a saturated-KCl-filled reference microelectrode (a sharp-ended microelectrode, see Section 4.1) is used to measure the electrical potential of solutions A and B in order to calculate corrections to the potentials measured by the ISM. Because K^+^ and Cl^−^ have equal mobilities, junction-potential errors introduced by the reference electrode are not a major concern. When the reference-electrode signals are subtracted from the measured ΔΨ*_solutionB_*_−_*_solutionA_*, we obtain the true ion-selective ΔΨ*_solutionB_*_−_*_solutionA_*. The reference microelectrode signal is also used to correct for differences in electrical potential between assay compartments. For example, if the ISM is used to impale a cell for intracellular measurements, the reference electrode must also be impaled into the cell so that Ψ does not include a contribution from the membrane potential of the cell (*i.e.*, the electrical potential difference between the cell cytoplasm and the extracellular compartment where the ISM was calibrated). The electrical configuration required to monitor the response of an ISM is represented in Figure 5.

In brief, the sample to be assayed (a solution or a cell in a solution) is placed in a recording chamber (such as those available from Warner Instruments). We use an FD223 dual-channel electrometer (World Precision Instruments Inc.) to monitor voltage signals. Both channels are connected to microelectrode holder/microelectrode assemblies via headstage probes that contain unity-gain amplifiers. The first probe is connected to an ISM and the second probe is connected to a saturated-KCl-filled reference electrode that senses the electrical potential of the assay space. Both probes should be mounted onto micromanipulators to allow fine positioning of the electrode tips.

Three features of the electrical set up allow accurate monitoring of the voltage signal from each electrode. Firstly, the amplifiers have an input-resistance/impedance (*Z_FD_*_223_ > 10^15^ Ω) that is many times greater than that of the electrode tip (for sharp-ended ISMs, *Z_ISM_* is typically ∼10^11^ Ω). This is an important consideration because the current that relays the input voltage signal (*V_in_*) from the assay space passes through the ISM tip as well as the amplifier and thus is influenced by the sum of their impedances (*Z_FD_*_223_ + *Z_ISM_*), whereas the current that relays the output voltage signal (*V_out_*) is influenced only by the impedance of the amplifier (*Z_FD_*_223_). A restatement of Ohm's law relates these quantities as:

(19) $$V_{\text{out}}=V_{in}\times \frac{Z_{FD223}}{Z_{\text{ISM}}+Z_{FD223}}.$$

Thus, the relatively high impedance of the amplifier compared to that of the ISM tip minimizes the loss of signal amplitude by ensuring that both *V_in_* and *V_out_* are influenced by total impedances of similar magnitude. Furthermore the higher impedance of the amplifier reduces phase shift which could distort the relationship between *V_in_* and *V_out_*.

Secondly, a signal-driven shield built into each probe, feeds a duplicate *V_in_* signal into the cable shielding, a maneuver that prevents stray capacitance between the signal-carrying wire and cable-shielding, increasing the responsiveness of the electrode. The shield also reduces electrical noise.

Thirdly, the dual-channel electrometer generates an ion-selective potential signal by subtracting the reference electrode signal (*V*_ref_) from the ISM signal (*V*_ISM_) with a high common-mode rejection ratio, which effectively eliminates noise common to both channels such that the true differential (*i.e.*, ion-selective) signal is accurately obtained, as depicted in Figure 5.

Note that, for intracellular recordings, the reference-electrode signal can be used to monitor membrane potential (*V*_m_) by clamping the electrical potential of the recording chamber to 0 mV using voltage-clamp circuitry (e.g., the OC-725C Oocyte Clamp from Warner Instruments), in which case *V*_m_ = *V*_ref_. Alternatively the potential of the extracellular space (*V*_chamber_) can be monitored with a calomel electrode or a second saturated-KCl-filled microelectrode (connected to a separate amplifier) and subtracted from *V*_ref_ using a separate subtraction amplifier, in which case *V*_m_ = *V*_ref_ − *V*_chamber_. Note that *V*_ref_ = *V*_chamber_ when the reference electrode is not impaled into a cell.

### 5.3. Calibration Procedure

The theory that underlies the calibration of ISMs is presented in Sections 2.5 and 5.2. An example calibration plot for a Na^+^-selective microelectrode is presented in Figure 2. A calibration slope of 58 mV/decade tells us that the ISM is ideally ion-selective with respect to its target ion. We use in-house software to convert the dual-channel electrometer output into a measure of ion concentration.

The series of solutions used to calibrate an ISM should have a composition that is close to that of the experimental sample to be measured (including potentially interfering ions), should cover the entire range of values expected to be encountered during the experiment, and should differ only in the concentration of target ion. For example, for calibration of a H^+^-selective ISM, we use a pH 6 buffer, a pH 7.5 buffer, and a pH 8 buffer. For ISMs that are less-ideally ion selective, such as Na^+^-selective microelectrodes, more calibration points may be necessary (e.g., a five-point calibration is shown in Figure 2).

## 6. Applications of ISMs

ISMs are suitable for obtaining both intracellular and extracellular measurements and can be used to monitor ion activities at a specific locus or, by virtue of self-referencing/vibrating probe technology (reviewed in references [3] and [32]), between two loci in order to gather information about ion gradients and fluxes. In this section we briefly review a selection of physiological studies that demonstrate the usefulness of ISMs.

The original application of the H^+^-selective (ionophore-based) ISMs reported by Ammann and coworkers was determination of the intracellular pH (pH_i_) of a *Xenopus* (frog) oocyte [16]. Cicirelli and coworkers extended this application to the study of changes in oocyte pH_i_ during oocyte maturation [33]. Others have harnessed H^+^-selective ISMs to monitor the activities of heterologously-expressed H^+^-coupled transporters in oocytes. For example, these studies have been critical to understanding the molecular physiology of the monocarboxylate (H/lactate) cotransporter MCT1 [34] and the H^+^-coupled oligopeptide transporter PepT1 [35]. Of course, the same usefulness applies to any other ion-selective ISM, for example the use of a Na^+^-selective ISM to characterize a Na^+^-coupled transporter. The observation of ion-movement across the plasma membrane is critical to distinguish ion-transport from ion-dependence (e.g., a demonstration of increased lactate transport by MCT1 at low extracellular pH is not the same as a demonstration that H^+^ is cotransported with lactate) and net ion-transport from gross ion-transport (e.g., see reference [26]).

H^+^-selective ISMs are also routinely used to monitor the other processes that affect the pH_i_ of cells. For example, the action of the electrogenic Na/HCO_3_ cotransporter NBCe1 (because HCO_3_^−^ uptake consumes H^+^ and raises pH_i_ [36]), the movement of CO_2_ across a cell membrane (because CO_2_ is hydrated as it enters the cell, generating H^+^ and lowering pH_i_ [37]), the action of the NH_4_^+^/NH_3_ channel AmtB [38], and the action of carbonic anhydrase II (that catalyzes the reversible hydration of CO_2_ [37]). Because the measured signals are robust, H^+^-selective ISMs can also be applied to the comparison of the activities of wild-type and mutant proteins expressed in oocytes. For example, ISMs have been used by multiple groups to study the molecular defects in Na^+^ and HCO_3_^−^ transport by disease-associated mutants of human NBCe1 [39–41].

ISMs can also be applied to measure changes in extracellular ion activities around cells and in intact tissues (e.g., those associated with swelling activated channels in epithelial cells [42] or neuronal activity in hippocampal slices from rats [43,44]), and changes in extracellular ion activities in the brains of whole animals (e.g., anaesthetized rats [45] and flies [46]). The study of intracellular ion activities in cells that may only have a diameter of 10 μm, requires the use of smaller tip-diameter ISMs, with *t*_90_ values that are on the order of tens of seconds. Because it is technically difficult to impale a small cell with both an ISM and a reference electrode, these studies are typically performed with double-barreled electrodes that combine the ISM and reference in a single tip. These have been used to monitor changes in intracellular ion activities in cells of isolated perfused tissues such as rabbit proximal tubules [25,47,48], sheep cardiac Purkinje fibers [49], and insect Malpighian tubule cells [50].

In the extracellular milieu, small changes in ion activity that result from ion channel or transporter activity can be difficult to reliably detect because local ion gradients at the transport site are quickly dissipated, especially if the cells are being superfused. In order to facilitate these measurements, investigators have developed the ion-trap technique [51–53] that uses a blunt-tipped ISM (Section 4.1) pushed up against the surface of a cell in order to isolate a small volume between the transport site and the ionophore cocktail within which a small accumulation or depletion of an ion will produce a substantial change in ion activity. This technique has been applied to the monitoring of CO_2_ and NH_3_ movement across the oocyte plasma membrane [54], the export of Cl^−^ by anion exchangers in the presence of HCO_3_^−^ [26], and the influx of H^+^ mediated by the Na^+^/glucose cotransport SGLT1 in the absence of Na^+^ [55].

As mentioned earlier, ISMs—with the exception of those based on H^+^-selective ionophores—are not overwhelmingly selective towards their intended target ion. However, this phenomenon can be used to an investigator's advantage. For example, the poor selectivity of some primitive “K^+^-selective” ISMs with respect to quaternary ammonium ions (such as the cell-impermeant cation tetramethylammonium, TMA^+^) means that these ISMs can be exploited to monitor cell volume regulation in cells loaded with TMA^+^ [56]. Underlying this application is the assumption that, if the intracellular TMA^+^ content is fixed, changes in measured TMA^+^ activity are due to changes in cell water content [56]. Another example of an “off-target’ use of an ISM relates to the “Na^+^-selective” ISM discussed in Section 3.2. These Na^+^-selective ISMs are actually somewhat more selective for Li^+^ than Na^+^. Because Li^+^ is not a major component of physiological solutions, Li^+^ interference with the ISM is not a usual consideration. However, in experiments conducted in the presence of extracellular Li^+^ and absence of extracellular Na^+^ an intracellular “Na^+^-selective” ISM can be used to monitor Li^+^ import across the plasma membrane, assuming that intracellular Na^+^ activity remains constant [57].

## 7. Outlook

The application of ISMs to the measurement of ion activities remains limited by the small size of individual cells in relation to the diameter of the ISM tip, which is why ISMs lend themselves better to use in large cells or tissue slices. Studies of ion perturbations in small, individual cells predominantly rely on the use of ion-sensitive dyes. In order to overcome size limitation, exciting efforts are currently underway to create miniaturized ISMs, and arrays of ISMs, by harnessing microfluidics technology (reviewed in reference [58]). Furthermore, ion-selective cocktails with novel and improved characteristics and ion-selectivities are constantly in development (e.g., [59–61] and review of ion-selective electrodes based on PVC-membranes that could be incorporated into ISMs in reference [62]), broadening the range of studies to which ISMs can be applied.

## Figures and Tables

**Table 1.** The composition and components of H^+^-selective ionophore I cocktail B (Sigma cat. no. 95293) and associated backfill solution. CAS (Chemical Abstracts Service) Registry numbers are provided for uncommon chemical components. Further details are provided at The Sensor Application Portal hosted at www.sigmaaldrich.com.

**Component**		**Composition**	**Requisite Characteristics**
Cocktail	Ionophore	10% (w/w) tridodecylamine (TDDA; CAS no. 102-87-4)	TDDA is a lipophilic amine that is predominantly uncharged in an organic solution equilibrated with a neutral aqueous solution, making it a neutral proton carrier [16].
	Solvent	89.3% 2-nitrophenyl octyl ether (*o*-NPOE; CAS no. 37682-29-4)	
	Additive	0.7% potassium tetrakis(4-chlorophenyl) borate (KTCPB; CAS no. 14680-77-4)	Reduces anion interference and electrical resistance without compromising ion-selectivity [16,18].
Backfill		40 mM KH_2_PO_4_, 15 mM NaCl, pH 7.0 with 23 mM NaOH [16]	Buffered electrolyte solution

**Table 2.** The composition and components of Na^+^-selective ionophore I cocktail A (cat. no. 99314, Sigma) and associated backfill solution. Further details are provided at The Sensor Application Portal hosted at www.sigmaaldrich.com.

**Component**		**Composition**	**Requisite Characteristics**
Cocktail	Ionophore	10% (w/w) N,N′,N″-Triheptyl-N,N′,N″-trimethyl- 4,4′,4″-propylidynetris(3-oxa- butyramide) (CAS no. 61183-76-4)	Forms a structure with a Na^+^ co-ordinating site that is relatively selective over intracellular interfering ions in the intracellular space (e.g., K^+^).
	Solvent	89.5% *o*-NPOE	
	Additive	0.5% sodium tetraphenyl borate (NaTPB; CAS no. 143-66-8)	Reduces anion interference, and electrical resistance without compromising ion-selectivity [18,19].
Backfill		10 mM NaCl	Contains no interfering ions.

**Table 3.** The composition and components of K^+^-selective ionophore I cocktail A (Sigma cat. no. 60031). Further details are provided at The Sensor Application Portal hosted at www.sigmaaldrich.com.

**Component**		**Composition**	**Requisite Characteristics**
Cocktail	Ionophore	5% (w/w) valinomycin (CAS no. 2001-95-8)	Valinomycin forms a ring structure that selectively co-ordinates K^+^[22].
	Solvents	25% 1,2-dimethyl-3-nitrobenzene (CAS no. 83-41-0)	
		68% dibutyl sebacate (CAS no. 109-43-3)	
	Additive	2% KTCPB	Contributes to cation-sensing, reducing anion-interference and reduces electrical resistance without compromising ion-selectivity [16,18].
Backfill		10–100 mM KCl [23,24]	Contains no interfering ions.

**Table 4.** The composition and components of Cl^−^-selective ionophore I cocktail A (Sigma cat. no. 99408). Further details are provided at The Sensor Application Portal hosted at www.sigmaaldrich.com.

**Component**		**Composition**	**Requisite Characteristics**
Cocktail	Ionophore	5% (w/w) *m*-Tetraphenyl-porphyrin manganese(III)-chloride complex (CAS no. 32195-55-4)	Ring structure that co-ordinates Mn^3+^, which has a greater affinity for Cl^−^ than for HCO_3_^−^, the other major physiological anion, conferring a useful selectivity to the cocktail [25,27,28].
	Solvents	90% *o*-NPOE	The addition of decanol reduces the electrical resistance of the cocktail, and increases its selectivity but at the cost of a reduced response time [25].
		4% decanol (CAS no. 112-30-1)	
	Additive	1% Tetradodecylammonium tetrakis(4-chlorophenyl)-borate (CAS no. 100582-42-8)	Reduces electrical resistance without compromising ion-selectivity.
Backfill		100 mM NaCl buffered with 10 mM Tris, pH 7.4 with H_2_SO_4_ [25].	Buffered Cl-containing solution that lacks interfering anions (divalent anions do not substantially interfere with porphyrin-based ionophores).
